# Ca^2+^ protein alpha 1D of CaV1.3 regulates intracellular calcium concentration and migration of colon cancer cells through a non-canonical activity

**DOI:** 10.1038/s41598-017-14230-1

**Published:** 2017-10-27

**Authors:** Yann Fourbon, Maxime Guéguinou, Romain Félix, Bruno Constantin, Arnaud Uguen, Gaëlle Fromont, Laurie Lajoie, Christophe Magaud, Thierry Lecomte, Emmanuel Chamorey, Aurélien Chatelier, Olivier Mignen, Marie Potier-Cartereau, Aurélie Chantôme, Patrick Bois, Christophe Vandier

**Affiliations:** 1 Inserm, UMR 1069, Université François Rabelais Tours, Tours, France; 2 Equipe ERL 7368 CNRS, Université de Poitiers, Poitiers, France; 3 Inserm UMR 1078 IFR148, Université de Bretagne Occidentale, Brest, France; 4 CHRU Brest, Brest, France; 5 GICC– UMR 7292 Université de Tours, Tours, France; 6 Unité d’Epidémiologie et Biostatistiques (UEB), Centre Antoine Lacassagne, Nice, France; 7 CHRU Tours, Tours, France; 8 Network “Ion channels and cancer-Canceropole Grand Ouest, (IC-CGO), Grand Ouest, France; 9 Present Address: Department of Cellular and Molecular Physiology, Penn State University School of Medicine, Hershey Medical Center, Hershey, PA 17033 USA

## Abstract

It is generally accepted that voltage-gated Ca^2+^ channels, CaV, regulate Ca^2+^ homeostasis in excitable cells following plasma membrane depolarization. Here, we show that the Ca^2+^ protein α1D of CaV1.3 channel is overexpressed in colorectal cancer biopsies compared to normal tissues. Gene silencing experiments targeting α1D reduced the migration and the basal cytosolic Ca^2+^ concentration of HCT116 colon cancer cell line and modified the cytosolic Ca^2+^ oscillations induced by the sodium/calcium exchanger NCX1/3 working in its reverse mode. Interestingly, NCX1/3 regulated membrane potential of HCT116 cells only when α1D was silenced, and blocking NCX1/3 increased cytosolic Ca^2+^ concentration and cell migration. However, membrane depolarization did not induce an increase in intracellular Ca^2+^. Patch-clamp experiments clearly showed that the inward Ca^2+^ current was absent. Finally, flow cytometry and immunofluorescence studies showed that α1D protein was localized at the plasma membrane, in cytosol and cell nuclei. Altogether, we uncover a novel signaling pathway showing that α1D is involved in the regulation of Ca^2+^ homeostasis and cell migration by a mechanism independent of its plasma membrane canonical function but that involved plasma membrane Na^+^/Ca^2+^ exchanger.

## Introduction

Voltage-gated Ca^2+^ channels (CaV) are activated by membrane depolarization and mediate Ca^2+^ influx in response to action potentials and subthreshold depolarizing signals. CaV are structurally made of five subunits (α1, α2, δ, β, γ) with the α1 subunit forming the pore of the channel^1^. The amino acid sequence is organized in four repeated domains each containing six transmembrane segments (S1–S6), and a membrane-associated loop between transmembrane segments S5 and S6^1^. It is generally accepted that CaV control Ca^2+^ homeostasis of excitable cells (such as cardiomyocytes, neurons, smooth and skeletal muscular cells) whereas non voltage-gated Ca^2+^ channels regulate Ca^2+^ homeostasis of non-excitable cells. Among CaV, CaV1.3 was found to be required for hearing^2^, for controlling excitability of chromaffin cells^3^ and for cardiac excitability by contributing either to diastolic depolarization of sino-atrial node pacemaker cells^4^ and atrial excitability^5^. In pathological conditions, CaV1.3 was found to contribute to the death of dopaminergic neurons in patients with Parkinson’s disease^6^ and to primary aldosteronism leading to arterial hypertension^7^.

Recently, meta-analyses showed that expression of genes encoding CaV subunits are increased in various cancers^8^. This was the case of the *CACNA1D* gene coding for the α1D subunit of CaV1.3 which is overexpressed in prostate, uterus and colon cancer^8^. In parallel, the α1D subunit was found to control the migration and the proliferation of endometrial cancer cells *via* the regulation of its expression by estrogens^9^ and the expression of androgens receptor in the prostate cancer cells^10^. Nevertheless, the biological role of the α1D subunit in colon cancer cells is not known, and it is important to recognize that it overexpression in colon cancer does not represent a causal link between high levels of the α1D protein and colon cancer. Colorectal cancer (CRC) represents major problems of public health because of its incidence and its mortality^11,12^. CRC is the third most common cancer in men (746,000 cases, 10.0% of the total) and the second in women (614,000 cases, 9.2% of the total) worldwide^13^. In France CRC is the second leading cause of cancer death and it accounts for nearly 12% of all cancer deaths, especially among those 65 years and older (http://www.e-cancer.fr/). In addition, CRC is characterized by metastasis development, which is the major cause of death of the patients developing a cancer. Although the mechanisms implied in the metastatic process are not yet completely elucidated, it is clear that the degradation of the extracellular matrix and the cellular migration, both regulated by Ca^2+^ channels^14^, play a pivotal role in this process.

It has not been determined if the regulation of the biology of cancer cells by α1D protein subunit depends on its plasma membrane canonical function. Indeed, α1D protein of CaV1.3 also has non-canonical functions and is involved in transcriptional regulation of the expression of other proteins including potassium channels (for review^15^). Indeed, α1D protein does not only control the activity of the Ca^2+^-activated K^+^ channel, SK2, of atrial cells but also its expression and its membrane localization. The C terminus of α1D protein translocates to the nucleus where it functions as a transcriptional regulator to modulate the function of SK2 channel^10^. In addition, the alternative splicing of C terminus of α1D protein, besides modifying the activity of CaV1.3, affects the pharmacological properties of CaV1.3 and its sensitivity to the DHP^16^.

Here we investigated the role of the α1D protein of CaV1.3 in the migration of the non-excitable and epithelial cancer cells HCT116, its contribution in intracellular Ca^2+^ regulation and we raised the question on its role as a channel in these cells. Our studies show that α1D protein is overexpressed in CRC biopsies compared to normal tissues. Alpha 1D protein regulates the migration and invasion of HCT116 colon cancer cells and its intracellular Ca^2+^ concentration by a mechanism that does not depend on its plasma membrane canonical function but that involves plasma membrane NCX1/3 exchanger and endoplasmic reticulum (ER) Ca^2+^ release.

## Results

### Cav1.3 is overexpressed in colon tumor biopsies

**Figure 1:**
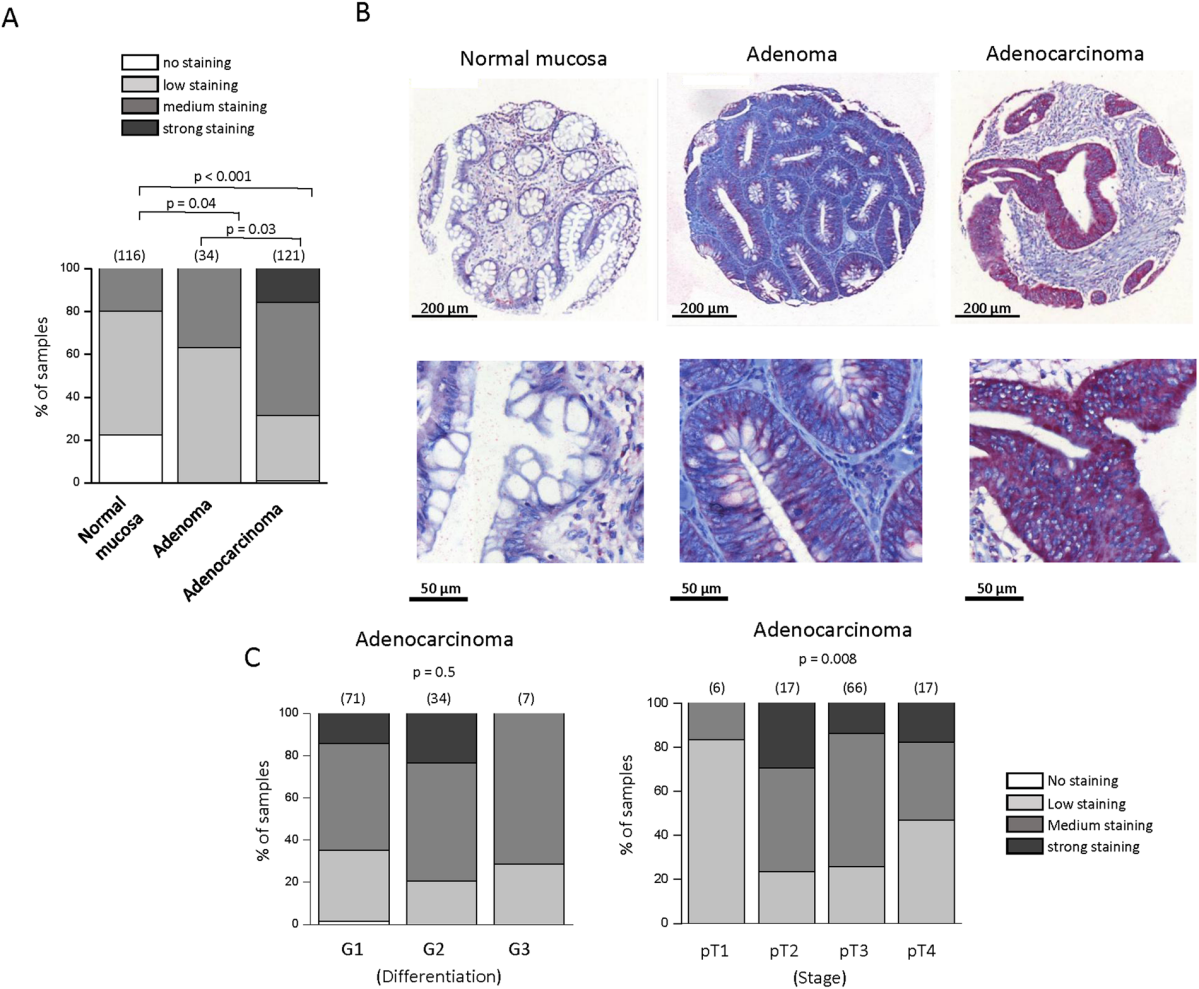
Immunohistochemical analysis of α1D protein expression in colon tissues microarrays. (**A**) Proportion of samples showing no (white), low (light gray), medium (black grey) or strong (black) α1D staining in normal mucosa, compared with adenoma and adenocarcinoma samples. The number of samples per condition is indicated in brackets. α1D staining was stronger in adenoma (*χ* ^2^, *p* < 0.04) and adenocarcinoma tissues (*χ* ^2^, *p* < 0.001) compared to normal tissues and in adenoma tissues compare to adenocarcinoma tissues (*χ* ^2^, *p* < 0.03). (**B**) α1D protein of CaV1.3 expression in normal mucosa (left), adenoma (center) and adenocarcinoma (right) from a same sample (1:100 dilution, Red revelation, Hematoxylin counter-coloration). Magnification, ×100 (top), ×300 (bottom). (**C**) Proportion of samples showing no (white), low (light gray), medium (black grey) or strong (black) α1D staining according to the state of differentiation (right) or stage (left) of adenocarcinoma samples. No difference was observed according the state of differentiation of adenocarcinoma samples (Kruskall Wallis test, p = 0.5). An influence of pT stages was observed on α1D staining (Kruskall Wallis test, p = 0.008).

We first explored the expression of α1 of CaV1 channels: α1S (α subunit of CaV1.1), α1C (α subunit of CaV1.2), α1D (α subunit of CaV1.3) and α1F (α subunit of CaV1.4) in CRC patients using the Human Protein Atlas (ref.^17^; www.proteinatlas.org). Among all α1 proteins only α1D was found overexpressed in CRC tissues compared to healthy tissues. Immunohistochemistry assays were performed on a series of 200 tissue-microarrays (TMA) included formalin-fixed and paraffin-embedded samples with paired tumor areas and healthy mucosa. α1D staining was stronger in adenoma and adenocarcinoma tissues compared to normal tissues and in adenoma tissues compared to adenocarcinoma tissues (Fig. 1A,B). There was no difference in staining between moderately/poorly-differentiated (i.e. G2/G3) adenocarcinomas and well-differentiated (*i.e*. G1) ones (Fig. 1C). We observed an influence of pT stages on α1D staining suggesting a role of α1D in the cell migration/invasion known to play a pivotal role in metastatic process (Fig. 1C).

### α1D promotes migration of HCT116 colon cancer cells

**Figure 2:**
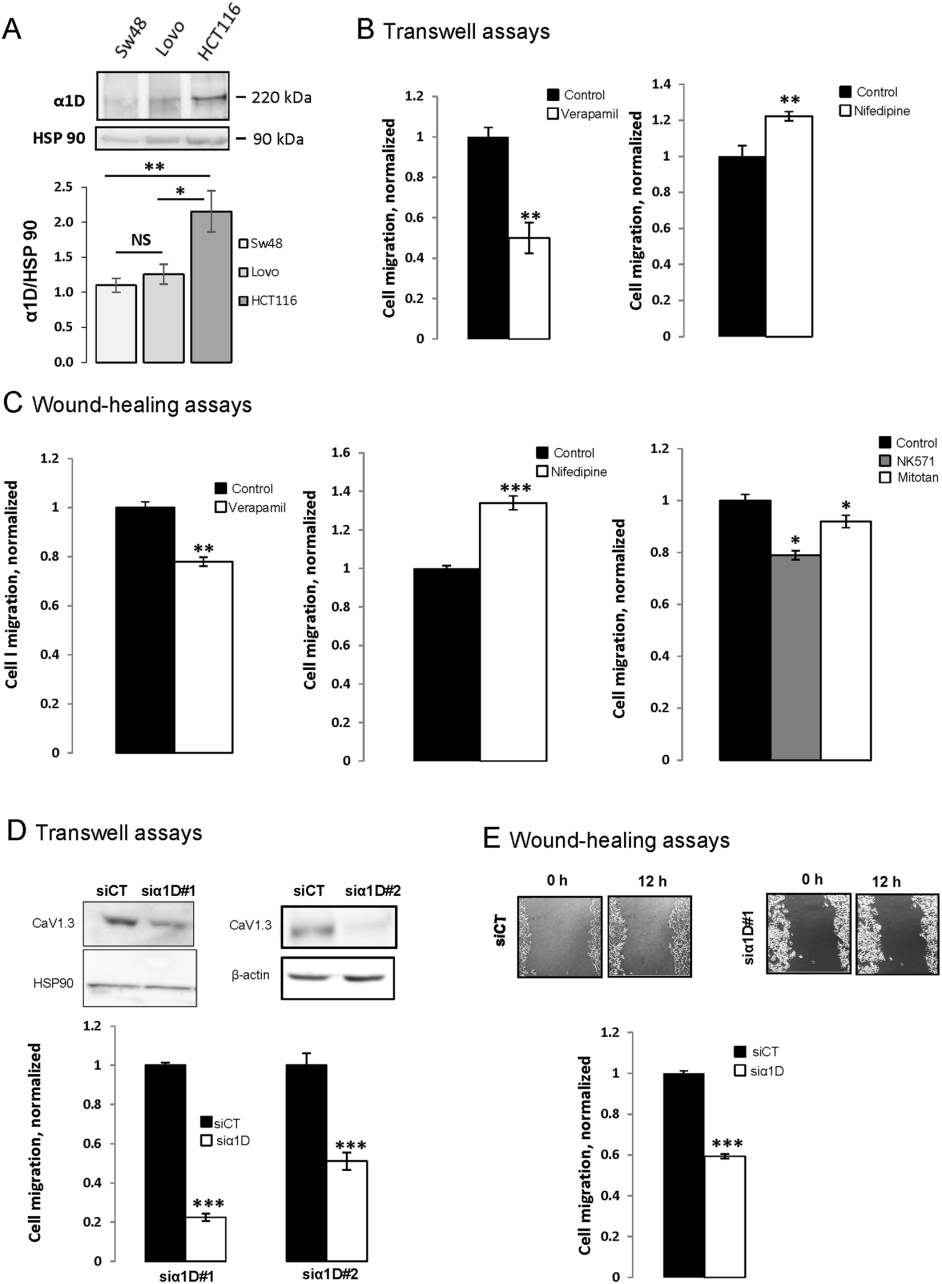
α1D protein regulates HTC116 cancer cell migration. (**A**) Representative cropped western blot of α1D protein expression in colon cancer cell lines (N = 3). Full-length western blots are included in the Supplementary Fig. 2A. Lower panel, levels of α1D proteins in colon cancer lines were determined by densitometry scanning to generate the values shown in the bar graph. Results are expressed as mean ± SEM. *p < 0.05 and **p < 0.01, (N = 3, Holm-Sidak test). (**B**) Histograms showing the effect of 10 µM verapamil and 10 µM nifedipine on HCT116 cell migration using inserts cell migration assays. The normalized cell number corresponds to the ratio of total number of cells in presence of drugs/total number of migrating cells in control experiments. Results are expressed as mean ± S.E.M. *Significantly different from control (p = 0.01, N = 3, n = 3, Mann Whitney test). (**C**) Histograms showing the effect of 10 µM verapamil (left), 10 µM nifedipine (middle) and 25 µM NK571 or 5 µM Mitotane (MDR blockers) (right) on HCT116 cell migration ability using a monolayer wound-healing assay. Results are expressed as mean ± S.E.M. *Significantly different from control (nifedipine: p = 0.001, N = 3, n = 9, verapamil: p = 0.005, N = 4, n = 12, NK571: p = 0.028, N = 3, n = 9 and Mitotane: p = 0.098, N = 3, n = 9, Mann Whitney tests). The normalized cell number corresponds to the ratio of capacity to repair the damaged area in presence of drugs/total capacity to repair in control. Effect of silencing α1D on migration of HCT116 using inserts (**D**) and wound-healing (**E**) cell migration assays. The inset shows the validation of α1D protein extinction by cropped immunoblots 48 h after transfection. Full-length Western blots are included in the Supplementary Fig. 2B. Results are normalized to si control condition and expressed as mean ± S.E.M. *Significantly different from control (inserts assays, p = 0.001, N = 3, n = 9, wound healing, p = 0.001, N = 4, n = 12, Mann Whitney tests). The image in E represent denuded areas at 0 and 12 h for cells silenced or not for α1D#1.

After analysing the expression of α1D in different tested CRC cell lines we investigated the role of α1D of CaV1.3 channel in the migratory ability of CRC cells. The effect of three well-known CaV blockers from three different pharmacology families, verapamil, nifedipine and diltiazem were tested on CRC cells. Figure 2A showed that α1D was found to be expressed in SW48, LoVo and HCT116 cell lines with a higher expression in HCT116 cell line.

Interestingly, HCT116 originates from a cancer classified as Duke D with higher metastatic potential than Duke C tumors, from which SW48 and LoVo lines are derived^18^. We therefore selected HCT116 cell line for subsequent experiments. Verapamil treatment at 10 µM (but not at 1 µM) decreased the number of migrating cells (transwell assay) by 50% and the viability of HCT116 cells by 25% (Fig. 2B, Supplemental Fig. 1A,B). In contrast, nifedipine at 10 µM (but not at 1 µM or 5 µM) increased the migration of HCT116 cells while having no effect on cell viability of HCT116 (Fig. 2B, Supplemental Fig. 1A,B). Finally, 10 µM diltiazem (another CaV blocker) has no effect on HCT116 cell migration (Supplemental Fig. 1B). To exclude that the decrease in cell migration by verapamil was caused by its effect on cell proliferation, cell migration was also assessed by a monolayer wound-healing assay. Cultures of confluent cells were scratched to create a denuded area, and then the cells at the wound edges were allowed to migrate into the denuded area over a 12 h period (during this time cells did not proliferate). After 12 h, the scraped areas were reduced and as observed using migration inserts nifedipine increased migration of HCT116 cells while verapamil reduced it (Fig. 2C). Since verapamil is a well-known multidrug resistance blocker in cancer cells we tested the effect of two multidrug resistance blocker, NK571 and mitotane, on migration of HCT116 cells. Figure 2C shows that these MDR blockers both reduced the migration of HCT116 cells. To confirm the contribution of α1D protein to HCT116 cell migration/invasion, α1D mRNA were silencing in HCT116 cells with two different siRNA of α1D (siα1D#1, siα1D#2), or with a scrambled-siRNA as a negative control (siCT). Figure 2D and Supplemental Fig. 2 shows a marked suppression of α1D expression in cells after transfection with siRNAs, when compared with cells transfected with scrambled-siRNA. The knockdown of α1D markedly reduced the migration (Fig. 2D,E) and the invasion (Supplemental Fig. 1C,D) of HCT116 without affecting cell viability (Supplemental Fig. 1D). These results suggest that α1D protein promotes HCT116 cell migration/invasion and that pharmacological blockers of CaV have a probable non selective effect.

### HCT116 colon cancer cell lack detectable voltage-gated inward Ca^2+^ currents

**Figure 3:**
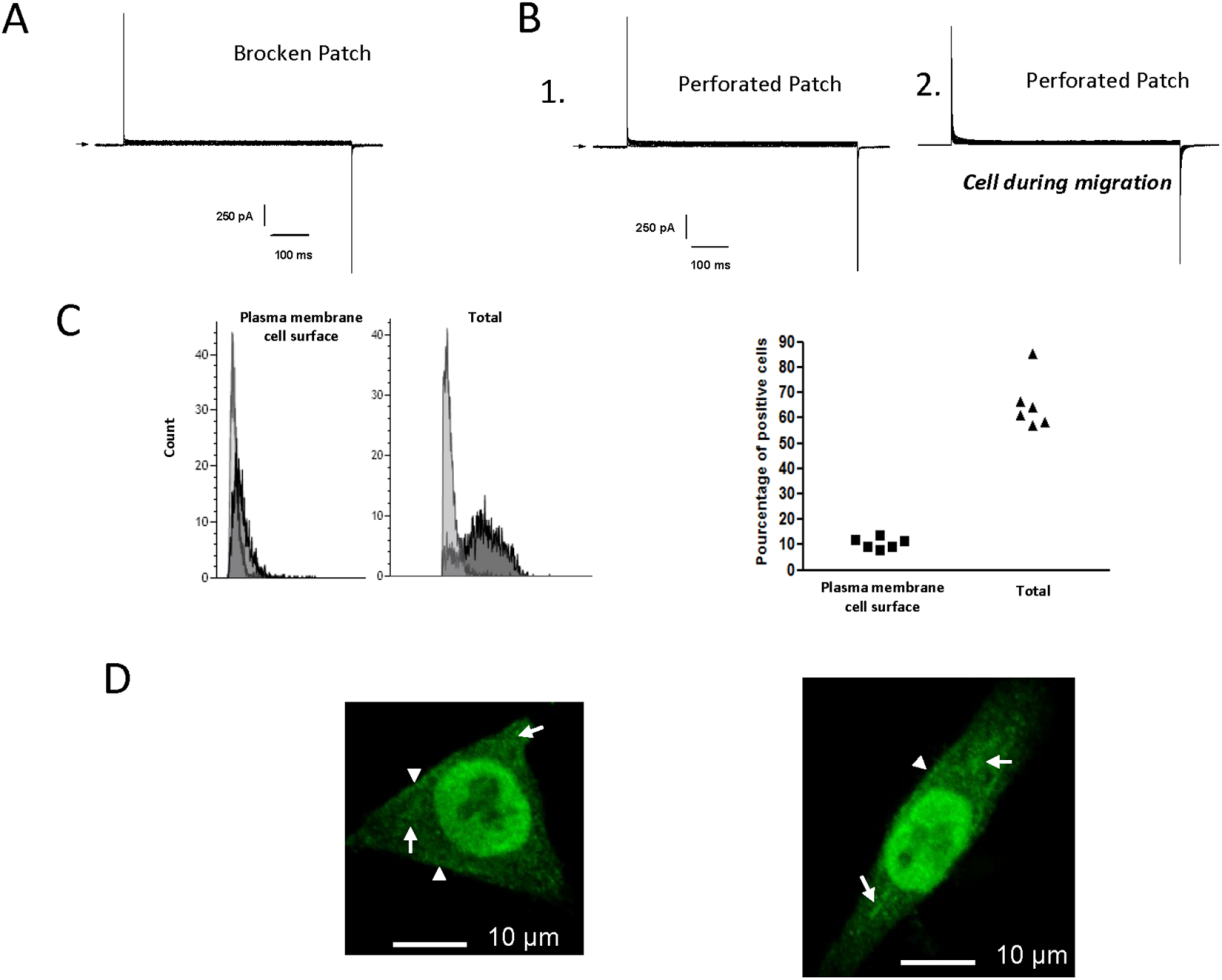
Lack of detectable voltage-dependent Ca^2+^ currents and cellular localization of α1D protein. (**A**,**B**) Current records during 600 ms pulses in the range of −70 to 110 mV from holding potential of −100 mV. Representative examples of current recording in presence of barium (20 mM) and Bayk 8644 (50 µM) in broken (**A**) and perforated (**B**) patch configurations. Whole cell currents are obtained in normal condition (B.1) and during the migration process (B.2). Arrows indicates zero current. (**C**) Fluorescence-activated cell sorter (FACS) analysis of α1D protein in HCT116 cell line permeabilized or not. Control staining is shown in light grey, and dark grey represent staining with anti-CaV1.3. Graph represents the percentage of cells (N = 6) expressing α1D protein in permeabilized (Total) and not permeabilized cells (Plasma membrane cell surface). (**D**) Subcellular distribution of **α**1D protein in HCT116 cell line. A primary anti-CaV1.3 antibody was used with a secondary AlexaFluor 488-conjugated anti-rabbit (green color). The short arrows indicate the location α1D protein in the edges membranes, and the long arrows show the presence of α1D protein in the cytosol.

The electrophysiological study was performed with the patch-clamp technique in whole cell configuration to test the canonical activity of α1D protein as CaV channel on the plasma membrane. To maximize the inward current conductance Ba^2+^ was chosen instead of Ca^2+^ and the CaV opener (Bay k 8644) was added (see methods). Figure 3A,B show currents record acquired in the range of −70 to 110 mV from holding potential of −100 mV. Surprisingly, no inward current was recorded in broken-patch configuration (Fig. 3A). Identically, no Ca^2+^ current was obtained in perforated-patch configuration, a configuration known to avoid rundown of the inward Ca^2+^ current (Fig. 3B1). This absence of inward Ca^2+^ current was obtained (in both experimental conditions) in 12 other cells. To test whether currents could be measured only in migrating cells, a cell monolayer in culture dishes was scratched, the migration of cells to the cleared area was inspected under a microscope 15 h after and cells were patched. Cell migration did not appear to affect the presence of Ca^2+^ current. Indeed, no inward Ca^2+^ current was recorded in migrating cells (Fig. 3B2, n = 5). The same results were obtained when cells were superfused with serum added during cell migration assays (Supplemental Fig. 1E). Previous studies have shown that cAMP/PKA pathway positively regulates CaV1.3 channels^3^. To study if the absence Ca^2+^ current is due to a lack of activation of basal cAMP/PKA pathway activation, 100 µM FSK (a well-known activator of adenylate cyclase) was applied to cells. In this condition no inward Ca^2+^ current was observed (Supplemental Fig. 1E). All together these experiments show that HCT116 cells lack detectable plasma membrane inward CaV currents.

### α1D protein is localized at the plasma membrane, in cell nuclei and cytoplasm

Having observed that α1D protein was necessary for cancer cell migration/invasion without a plasma membrane canonical function we assessed its cellular localization. To determine the cellular distribution of α1D protein, HCT116 cells were permeabilized or not and analyzed by FACS using an antibody recognizing an extracellular epitope of α1D protein when part of the CaV channel (Fig. 3C). The proportion of cells expressing α1D at the plasma membrane surface was low: as measured by fluorescence analysis of intact or permeabilized cells with an α1D protein antibody, ≈70% of cells showed entirely intracellular localization of α1D protein (Fig. 3C). To confirm these findings, immunofluorescence confocal microscopy studies were performed on permeabilized HCT116 cells. As suspected using the antibody that recognize the extracellular α1D epitope, a major proportion of α1D protein appeared to be localized in the cytoplasm and in the cell nuclei with a slight expression at the plasma membrane *(*Fig. 3D *)*. It was observed that the β3 subunit of CaV favors Ca^2+^ channel plasma membrane expression by facilitating the intracellular trafficking of α1D subunit toward the plasma membrane^19^. We observed a marked expression of the β3 subunit in HCT116 cells suggesting that its presence does not obligatory cause the plasma membrane localization of α1D protein (Supplemental Fig. 3). Another protein, calreticulin a protein complexing Ca^2+^ normally found in ER was found to negatively regulate the surface expression of Cav1.3^20^. Silencing of calreticulin did not result in increased this localization (Supplemental Fig. 4) demonstrating that this protein is not involved in intracellular retention of α1D protein.

### α1D protein regulates basal cytosolic Ca^2+^ concentration of HCT116 cells

**Figure 4:**
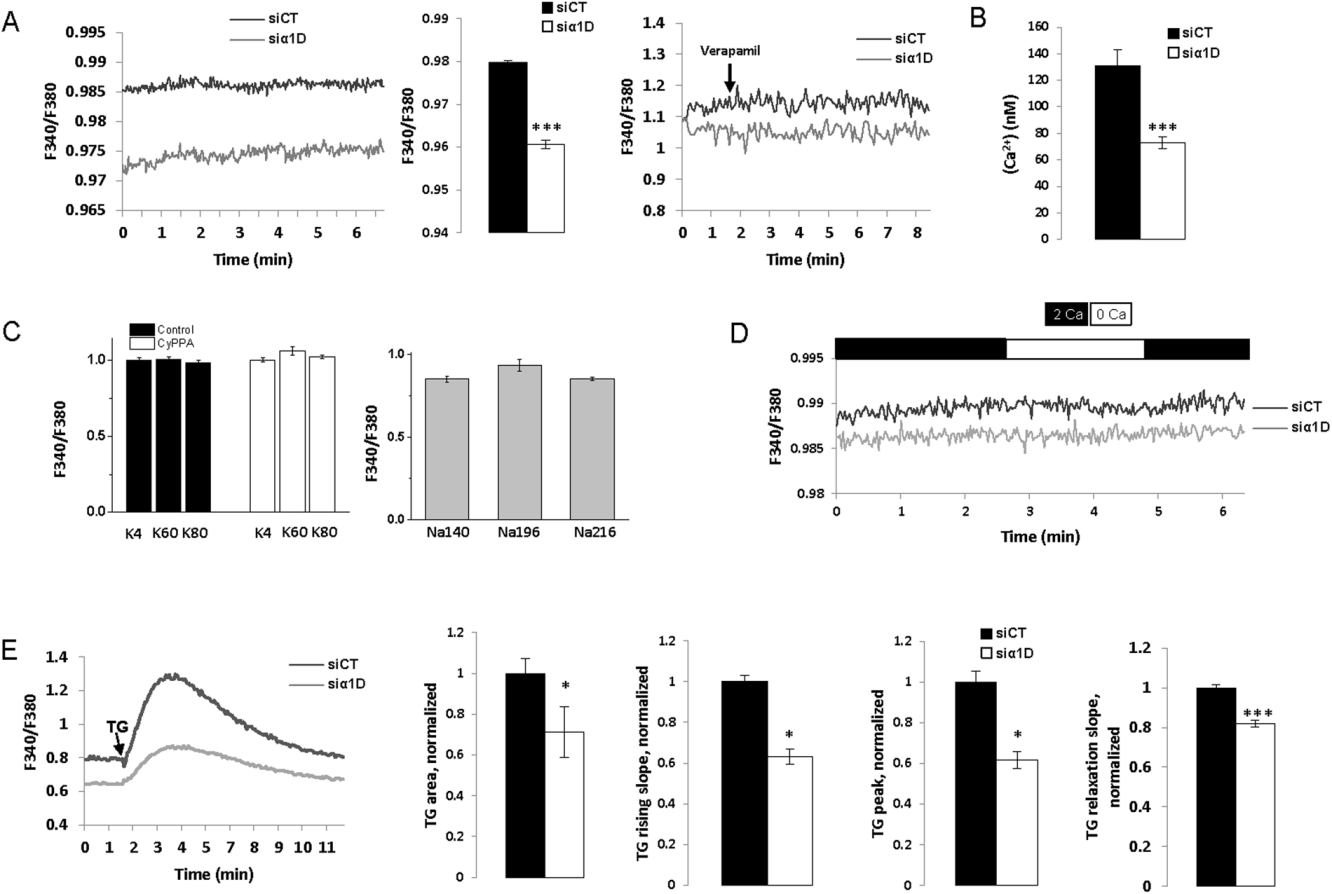
α1D protein regulated basal cytosolic Ca^2+^ concentration of HCT116 cells. (**A**) left, example of measurement of F340/F380 recorded in control condition and in cell in which α1D was silenced. Middle, histograms showing mean ± S.E.M of F340/F380 with a significant differences between control and siα1D#1 conditions (F340/F380: p = 0.001, N = 5, n = 89 si-control; N = 6, n = 97 siα1D, Mann Whitney tests). Right, representative traces recorded in the presence or not of verapamil in control condition and in cell in which α1D was silenced (N = 3, n = 6). (**B**) Histograms showing mean ± S.E.M of [Ca^2+^]_c_ with a significant differences between control and siα1D#1 conditions (p = 0.001, N = 3, n = 47 si-control; N = 3, n = 58 siα1D#1, Mann Whitney tests). (**C**) left, effect of high external concentrations of K^+^ (4 + 56 mM: K60 and 4 + 76 mM: K80) with or without 20 µM CyPPA on [Ca^2+^]_c_ (N = 3, n = 17) and of high external concentrations of Na^+^ as osmotic control solutions (140 + 56: Na196 and 140 + 76: Na216). None of the changes induced a significant effect (N = 3, n = 10). (**D**) Time dependent measurements of [Ca^2+^]_c_ with (2 Ca) or without (0 Ca) external Ca^2+^ solutions. (**E**) left, time dependent measurements of [Ca^2+^]_c_ in the presence of TG (5 µM) in control cells and cells that have been silenced for α1D (siα1D#1). Histograms showing the effect of silencing α1D on TG rising slope, the TG area, the TG peak Ca^2+^ responses and the TG relaxation slope. Results are normalized to siCT and are expressed as mean ± S.E.M. *Significantly different from control (p = 0.001, N = 4, n = 32, Mann Whitney test).

Our findings revealed a novel signaling pathway in which the intracellular α1D protein promoted cancer cell migration/invasion. Since cell migration/invasion has been found to be regulated by cytosolic Ca^2+^ concentration [Ca^2+^]_c_^14^, we tested the hypothesis that α1D protein regulated [Ca^2+^]_c_ despite its lack of plasma membrane channel activity. Figure 4A,B shows that silencing α1D mRNA reduced the F340/F380 ratio of fura-2 fluorescence, and [Ca^2+^]_c_ of HCT116 cells from approximatively 130 nM to 70 nM. In contrast, acute applications of 10 µM verapamil or 10 µM nifedipine had no effect on F340/F380 of HCT116 cells (Fig. 4A, Supplemental Fig. 3C). An increase in [Ca^2+^]_c_ is generally observed following depolarisation with high external K^+^ concentration in excitable cells when α1 subunit forms a CaV channel in the plasma membrane. Depolarization of the plasma membrane by increasing external K^+^ concentration up to 80 mM (Ek = −15 mV and activation threshold of CaV1.3 around −40 mV^21^) did not increase [Ca^2+^]_c_ (Fig. 4C). At −50 mV (the resting membrane potential of HCT116 that is regulated by SK3 channel^22^) a small steady-state inactivation of CaV1.3 channel had been observed^21^. To assay whether the absence of effect of elevated K^+^ concentrations on [Ca^2+^]_c_ was due to the inactivation of α1D protein we first hyperpolarized plasma membrane of HCT116 using the SK3 channel activator, CyPPA^23^, before increasing external K^+^ concentration. Figure 4C shows that despite of CyPPA pretreatment, an elevation of external potassium did not alter [Ca^2+^]_c_. In addition, 10 µM verapamil did not change [Ca^2+^]_c_, as recorded in cells exposed to 40 mM of external K^+^ (Supplemental Fig. 3B). α1D protein of CaV1.3 can regulate basal [Ca^2+^]_c_ either by activating a constitutive Ca^2+^ entry of Ca^2+^ from extracellular side or by promoting Ca^2+^ release from intracellular stores such as the ER. Figure 4D shows that the suppression of extracellular Ca^2+^ did not change basal [Ca^2+^]_c_ of HCT116 cells that had been treated with α1D siRNA. This suggests that α1D protein does not regulate basal [Ca^2+^]_c_ through a constitutive Ca^2+^ entry from plasma membrane. In contrast, the suppression of α1D protein reduced the thapsigargin (TG) Ca^2+^ responses of HCT116 cells by decreasing the rising TG slope, TG area, TG peak Ca^2+^ responses and the relaxation TG slope (Fig. 4E). The effect on TG response (i.e. decrease in peak, area and rising slope) suggests that α1D protein enhances Ca^2+^ ER release and/or [Ca^2+^] ER loading, by a mechanism independent on Ca^2+^ current through α1D. On the other hand, the effects of α1D protein on basal [Ca^2+^]_c_ and/or TG response (decrease in area and in relaxation slope) could be explained by regulation of PMCA Ca^2+^ efflux, Na^+^/Ca^2+^ exchanger (NCX) Ca^2+^ efflux or mitochondria Ca^2+^ uptake. Interestingly, silencing α1D had no effect on store operated Ca^2+^ entry (SOCE) of HCT116 cells (Supplemental Fig. 5).

### α1D protein regulates cytosolic Ca^2+^ concentration by inhibiting NCX1/3 and promoting ER Ca^2+^ release

**Figure 5:**
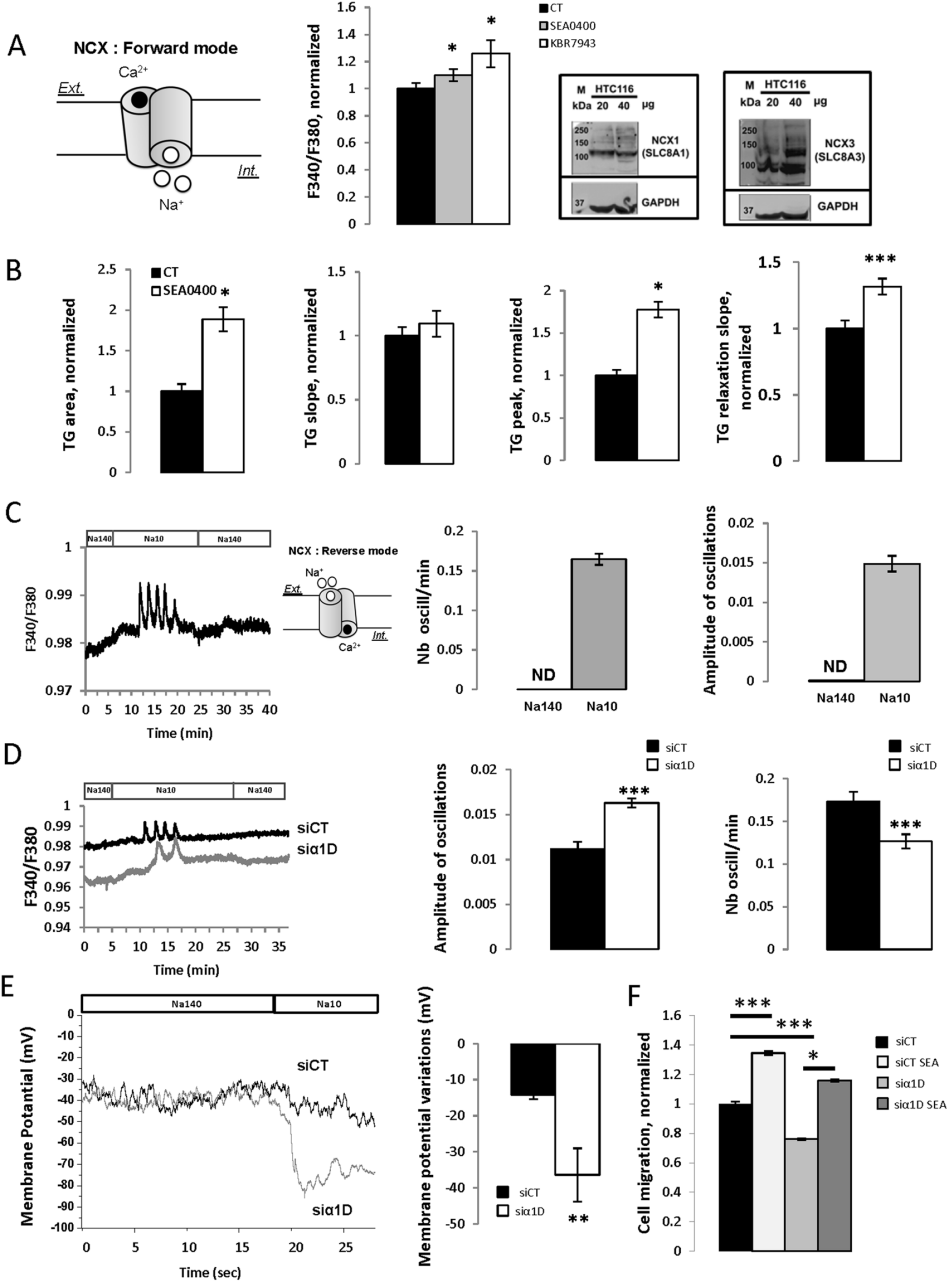
α1D protein regulated cytosolic Ca^2+^ concentration by inhibiting NCX. (**A**) left, the schema represents NCX working in its forward mode driving Na^+^ entry and Ca^2+^ efflux. Middle, Histograms showing the effect of 10 µM SEA0400 or 30 µM KBR7943 on F340/F380 ratios. Results are normalized to CT conditions and are expressed as mean ± S.E.M. *Significantly different from control (p = 0.05, N = 3, n = 12, Kruskal-Wallis one way analysis of variance. Right, Representative cropped Western blot of NCX1 and NCX3 in HCT116 colon cancer cells (N = 3). Full-length Western blots are included in the Supplementary Fig. 6. (**B**) Histograms showing the effect of 10 µM SEA0400 on TG slope, the TG area, the TG peak Ca^2+^ responses and the TG relaxation slope. Results are normalized to CT conditions and are expressed as mean ± S.E.M. *Significantly different from control (p = 0.001, N = 3, n = 12, Mann Whitney test). (**C**) Time dependent measurements of [Ca^2+^]_c_ in the presence of 140 mM (Na140) or 10 mM (Na10) of external Na^+^ concentration. The schema represents NCX working in its reverse mode driving Na^+^ efflux and Ca^2+^ entry. Histograms showing the effect of reducing external Na^+^ concentration to 10 mM on the amplitude and on the frequency of Ca^2+^ responses. Results are expressed as mean ± S.E.M. (**D**) Time dependent measurements of [Ca^2+^]_c_ in the presence of 140 mM or 10 mM of external Na^+^ concentration in control cells or in cells that have been silenced for significant differences between control and1D. Histograms showing the effect of silencing significant differences between control and1D (si significant differences between control and1D#1) on the amplitude and on the frequency of Ca^2+^ responses. Results are expressed as mean ± S.E.M. *Significantly different from control (amplitude: p = 0.001, N = 5, n = 77; N = 6, n = 94, Mann Whitney test and for frequency: p = 0.001, N = 5, n = 53; N = 6, n = 81, Mann Whitney test). (**E**), examples of membrane potentials recorded in one control HCT116 cell and in one HCT116 cell that have been silenced for α1D using the patch clamp technique in current clamp mode. Membrane potentials were recorded in Na140 or Na10 solutions. Histogram showing membrane potential variations between Na140 and Na10 conditions in control and significant differences between control and1D cells. Results are expressed as mean, bars, SEM. *Significantly different from control (p = 0.005, N = 3, n = 7 si-control; N = 3, n = 6 si significant differences between control and1D#1, Mann Whitney test). ND: not determined. F. Effect of silencing significant differences between control and1D in presence or not of 1 µM SEA0400 on migration of HCT116 cells (wound-healing cell migration assays). Results are normalized to si control conditions and results are expressed as mean ± S.E.M. *Significantly different from control (Si-Control with SEA0400; si significant differences between control and1D; si significant differences between control and1D with SEA0400, N = 4, n = 14).

In order to maintain low [Ca^2+^]_c_, NCX exchanges one Ca^2+^ ion for three Na^+^ ions. In its forward mode, inward (depolarizing) Na^+^ current drives Ca^2+^ extrusion from the cell while the reduction of Na^+^ current (by reducing external Na^+^ concentration) forces Ca^2+^ entry by NCX thus working in its reverse mode. Figure 5A shows that KBR-7943 and SEA0400, two NCX blockers, both increased basal [Ca^2+^]_c_ demonstrating that NCX drives Ca^2+^ extrusion of HCT116 cells. Western blot analysis of HTC116 extracts revealed a band of 110 kDa approximately, for NCX1 exchanger and bands of 110 to 140 kDa as expected for NCX3 exchanger (Fig. 5A). We did not detect NCX2 (data not shown). SEA0400 increased Ca^2+^ TG area and TG peak responses while having no effect on Ca^2+^ TG rising slope and increase the relaxation slope (Fig. 5B) suggesting that NCX1/3 regulates the decrease phase of [Ca^2+^]_c_ after TG- induced Ca^2+^ release. As expected, decreasing external Na^+^ concentration increased [Ca^2+^]_c_ and induced Ca^2+^ oscillations (Fig. 5C). Silencing α1D increased the amplitude of Ca^2+^ oscillations and reduced their frequency compared to siControl (Fig. 5D). As NCX1/3 are electrogenic it should regulate membrane potential of HCT116 cells and α1D should reduce its effect. We addressed this suggestion by applying current-clamp experiments to directly measure the effect of silencing α1D upon the regulation of cell membrane potential by NCX1/3. Compared to control cells (Em = −57 ± 5 mV, n = 7), silencing of α1D reduced the negative membrane potential (Em = −38 ± 6 mV) (p = 0.037), suggesting that when NCX1/3 works in its forward mode (with a depolarizing Na^+^ current) α1D reduced its activity and hyperpolarized cells to −57 mV. We next examined the ability of decreasing external Na^+^ concentration to hyperpolarize the cells. Figure 5E shows membrane potential records of control and siα1D cells in solutions containing 140 mM (Na140) and 10 mM Na^+^ (Na10) solutions with a significant higher hyperpolarization membrane potentials observed in cells with silenced α1D. Finally, SEA0400 markedly increased the migration of HCT116 suggesting that α1D protein promotes cell migration by inhibiting NCX1/3 (Fig. 5F). In addition, same results were observed with LoVo cells (Supplemental Fig. 7).

## Discussion

Our data have demonstrated that α1D protein regulates migration/invasion of HCT116 cells mainly through its non-canonical activity because: i) we did not detect any voltage-gated inward Ca^2+^currents in these cells using patch -clamp technique irrespective of the clamp configurations or the experimental conditions and ii) depolarization with high external K^+^ solutions did not increase the [Ca^2+^]_c_ in contrast to what had been observed in excitable cells.

α1D protein of CaV channels has been found expressed in various cancer forms^8^ and has both canonical and non-canonical functions (for review see^15^). It was already observed that α1D protein controls migration and proliferation of endometrial cancer cells^9^. In these cells, α1D protein expression was regulated by estrogen controlling Ca^2+^ influx^9^, and nifedipine was found to decrease cell migration, proliferation and autophagy^24^. *CACNA1D* (gene encoding α1D) was found to be overexpressed in prostate cancer^8^ and nifedipine or verapamil reduced androgen-stimulated [Ca^2+^]_c_ increase^10,25^. None of these reports have measured the Ca^2+^ channel activity of α1D protein using patch-clamp, the gold standard technique. Demonstrating that nifedipine/verapamil or knocking down α1D modified [Ca^2+^]_c_ and cell migration is not sufficient to conclude that α1D protein works as a Ca^2+^ channel. While diltiazem has no effect on cell migration, we found that nifedipine increased the migration capacity of HCT116 cells. Guo *et al*.^26^ reported that nifedipine stimulated the migration of cancer cells *via* the axis of miRNA-524-5p-BRI3–Erk pathway independently of its Ca^2+^ channel-blocking activity. Among non-selective effects of pharmacological inhibitors of CaV channels, nifedipine and verapamil were also found to inhibit potassium channels^27,28^ and we demonstrated that multidrug resistance blockers mimicked the effect of verapamil on cell migration.

All these data strongly suggest that the effects of verapamil and nifedipine on HCT116 cell migration are not related to their Ca^2+^ channel-blocking activities. Moreover, CaV channels are activated on membrane depolarization and we found that increasing external potassium concentration up to 80 mM did not change [Ca^2+^]_c_. This was not due to an inactivated state of CaV1.3 since with CyPPA, a SK3 channel activator regulating membrane potential of HCT116, [Ca^2+^]_c_ was not changed when increasing external potassium concentrations. All together, these data support a non-canonical function of α1D protein in the regulation of [Ca^2+^]_c_ and calcium oscillations of HCT116 cells.

α1D protein was found to regulate NCX of HCT116 cells. NCX genes comprise a family of three genes (NCX1, NCX2 and NCX3) thought to be involved in maintaining a [Ca^2+^]_c_^29^, with NCX1 and NCX3 found expressed in HCT116 colon cancer cells. At physiological extracellular concentration of Na^+^ (140 mM), NCX allows the outward flow of Ca^2+^ ^29^. This is what we observed in HCT116 cells with NCX1/3 working in their forward mode. α1D expression partly block NCX1/3, which can explain the relatively polarized values of membrane potential of these cells (NCX blockade by silencing α1D depolarized cells by 20 mV). Using two blockers of NCX (KB-R7943 and SEA0400) we demonstrated that NCX1/3 regulate [Ca^2+^]_c_ of HCT116 by driving Ca^2+^ extrusion out of cell. Moreover, blocking of NCX with SEA0400 increased the migration of the HCT116, probably by increasing [Ca^2+^]_c_. After switching to a low external Na^+^ solution an increase of [Ca^2+^]_c_ was observed with the generation of Ca^2+^ oscillations. This is explained by the exchanger working in its reverse mode allowing an inward flow of Ca^2+^, which increase of [Ca^2+^]_c_. In this reverse mode NCX1/3 hyperpolarized HCT116 cells. The suppression of α1D protein of CaV1.3 was found to reduced [Ca^2+^]_c_, TG area, TG peak Ca^2+^ responses and increased hyperpolarization, [Ca^2+^]_c_ and oscillations amplitudes following reduction of extracellular Na^+^ concentration. This suggests that α1D protein negatively regulates NCX1/3 working in its forward (inhibits outward flow of Ca^2+^) and reverse mode (inward flow of Ca^2+^). Intracellular Ca^2+^ oscillations has been observed in epithelial cancer colon cells^30^ and were found to play important roles in carcinogenesis^31^ but to our knowledge this is the first report showing a regulation of intracellular Ca^2+^ oscillations by α1D protein and NCX1/3. The molecular mechanisms regulated by NCX1/3 and CaV1.3 involved in [Ca^2+^]_c_ and Ca^2+^ oscillations remain to be elucidated but it seems unlikely that this is through the canonical function of plasma membrane CaV1.3. We postulate that α1D protein interacts directly or indirectly with NCX1/3 inducing the inhibition of NCX1/3. Such a direct interaction between NCX and transient receptor potential (TRP) was demonstrated by reciprocal co-immunoprecipitation and glutathione S-transferase (GST)-pulldown experiments in rat cardiac myocytes^32^. Moreover, interaction between α1D protein and NCX1/3 should be favored by their localization in caveolae and probably by the presence of specific protein and/or lipids in these lipid rafts like caveolin and cholesterol. Another possibility is that a particular non pore α1D protein, due to mutation or posttranslational modifications, may favor the interaction and the inhibition of NCX1/3. Moreover, α1D protein both from plasma membrane and ER can modulate plasma membrane NCX1/3. Interestingly, even with low external Na^+^ concentration, [Ca^2+^]_c_ was found to be lower after knocking down of α1D compare to control cells. This can be explained by a control of ER Ca^2+^ release and/or PMCA Ca^2+^ efflux and/or mitochondria Ca^2+^ uptake by α1D. We can speculate that this depends on the localization of α1D that was mainly found in intracellular compartments and probably ER. The cause of this particular localization remains to be elucidated.

In addition to regulate [Ca^2+^]_c_ through its non-canonical function α1D protein was found to work as a transcription factor regulating the expression of proteins involved in the regulation of [Ca^2+^]_c_ and cell migration. Indeed, a fragment of the CaV1.3 C-terminus was reported to be translocate to the nucleus where it regulates the expression of the Ca^2+^-activated K^+^ channel, SK2 channel, and protein regulating cell migration such as the myosin light chain^33^. The C-terminus of CaV1.2 also acted as a transcription factor and its overexpression altered the expression of NCX1, the Ca^2+^ channel TRPV4 and Ca^2+^-activated K^+^ channel, SK3 channel^34^. In addition, the C-terminal end of CaV1.3 would play a significant role in the sensitivity to antagonists of CaV1.3 particularly those of the family of dihydropyridines (ex: nifedipine). Indeed, the alternate splicing of distal C-terminal end of CaV1.3, besides modifying the activity of CaV1.3, affects the pharmacological properties of CaV1.3 and the sensitivity to the DHP^16^. Since we detected α1D protein in the nucleus further work needs to be done to confirm this data and elucidate the role of nuclear α1D in HCT116 cells.

In conclusion, our data have shown that α1D protein regulates the migration and invasion of HCT116 colon cancer cells and its intracellular Ca^2+^ concentration by a mechanism that did not depend on its plasma membrane canonical function but that involved plasma membrane NCX1/3 exchangers and ER Ca^2+^ release. In cancer, because of aberrant expression of Ca^2+^ channels, Ca^2+^ signaling becomes distorted and these alterations can cause a deregulation of Ca^2+^-dependent effectors that control signaling pathways determining cellular behavior and promoting pathophysiological cancer hallmarks in addition to decreasing chemotherapeutic efficacy. The discovery of new strategy aiming at decreasing one of these characteristics could have major repercussions in Public health. Consequently, the implication of α1D protein of CaV1.3 in the Ca^2+^-dependent migration of cancer cells and it overexpression in colorectal cancer patients represents an opportunity to consider a new therapeutic concept.

## Methods

### Cell line culture

Colons cancer cell lines HCT116, Lovo and SW48 were obtained from American Type Culture Collection (ATCC) and cultured in Opti-MEM supplemented with 10% fetal bovine serum (FBS), without antibiotics at 37 °C in 95% (v/v) air /5% (v/v) CO_2_. Ahmed *et al*., described disease stage, type, epigenetic and genetic features of these cancer cell lines that are derived from patients having colon cancer^18^.

### Immunohistochemistry

Tissue microarray (TMA) blocks were built on the basis of 200 formalin-fixed and paraffin-embedded colorectal samples (166 adenocarcinomas and 34 adenomas). The characteristics of patients and tissues are summarized in Table 1. Written informed consent was obtained from all patients and all samples were included in the registered tumor tissue collection n° DC-2008-214. Immunohistochemistry was performed on tissue section from the TMA blocks using CaV1.3 (HPA020215, Sigma Aldrich, Red revelation). Staining intensity was assigned with a semi-quantitative scale as follows: 0, no stained cells; 1, faint or weak staining; 2, moderate staining; or 3, strong staining intensity.

**Table 1:** Patients and tissues characteristics.

Groups	ADE (n = 34)	ADK (n = 165)
Age y, median (range)	62 (39–87)	69 (28–89)
Male/Female	24/10	107/58
Localization		
Rectum	n = 3	n = 44
Left colon	n = 22	n = 56
Transverse colon	n = 1	n = 8
Right colon	n = 6	n = 55
Differentiation		
G1		n = 91
G2		n = 48
G3	NA	n = 14
Pathological stage		
pTis	NA	n = 20
pT1		n = 7
pT2		n = 20
pT3		n = 96
pT4		n = 22

NT: normal colorectal tissue; ADE: adenoma; ADK: adenocarcinoma. y: years; NA, not applicable.

### Electrophysiological recordings

Currents were recorded using two whole-cell configurations; “broken patch” and “perforated patch”. The measurements were carried out at room temperature (22 °C). Fire-polished, patch electrodes (2 MΩ) were pulled from borosilicate glass capillaries using a vertical micropipette puller (Narishige, Tokyo, Japan). Voltage -clamp experiments were performed using an Axopatch 200 A amplifier with a CV 203BU headstage (Molecular Devices, Sunnyvale, CA, USA). Series resistance compensation was performed to values >80% to minimize voltage errors. Voltage command pulses were generated by a personal computer equipped with an analog-digital converter (Digidata 1200, Molecular Devices) using pCLAMP software v8.0 (Molecular Devices). To obtain the perforated patch configuration^35^, amphotericin B was added in the pipette solution (without EGTA). Amphotericin B was dissolved in dimethylsulfoxide (50 mg/ml) and diluted to a final concentration of 0.15 mg/ml in electrode solution. A gigaohm seal was established on the cell surface and capacitance transients were monitored. After the gigaseal between the pipette and the cell was achieved, the electrical access to the cytoplasm was monitored by applying 10 mV pulses for 10 ms from a holding potential of −70 mV and monitoring the capacitive transient.

The patch pipettes were filled with (mM): TEACl 120, MgCl_2_ 3.5, HEPES/NaOH 10, pH = 7.2. EGTA at 10 mM was added in normal whole-cell patch configuration. The bath solution contained (mM): CsCl 100, KCl 2.5, BaCl_2_ 20, and HEPES 10. The pH was adjusted to 7.4 using NaOH.

The use of Ba^2+^ as the charge carrier instead of Ca^2+^ has a number of advantages: (1) conductance for Ba^2+^ ions *versus* Ca^2+^ ions through Ca^2+^ channels is larger^36^, thereby increasing the signal-to-noise ratio; (2) in the presence of Ba^2+^ ions, the inactivation of L-type Ca^2+^ channel is slowed while the inactivation of the T-type is unaffected, which helps for their identification^37^; (3) it reinforces blocks to many K^+^ currents; and (4) Ba^2+^ was chosen instead of Ca^2+^ to suppress residual outward currents due to Ca^2+^ mediated permeability.

In voltage-clamp mode IV protocol was performed with a succession of depolarizing pulses from −70 to 50 mV with 20 mV steps and 600 msec duration from a holding potential at −100 mV. The current was filtered at 5 kHz and sampled at 50 kHz.

Current-clamp (I = 0) experiments were performed using the “broken patch” whole-cell recording configuration of the patch -clamp technique. Pipette solutions contained (in mM): K-glutamate 125, KCl 20, MgCl_2_ 1, Mg-ATP 1, HEPES 10, and pH was adjusted to 7.2 with KOH and various concentrations of CaCl_2_ and EGTA were added to obtained calculated pCa = 6 (0.87 mM CaCl_2_ and 1 mM EGTA) or pCa = 7 (0.37 mM CaCl_2_ and 1 mM EGTA).

### Intracellular Ca^2+^ measurement

Intracellular Ca^2+^ concentrations were estimated using the ratiometric fluorescent dye Fura-2. Cells were plated on cover slips (Fluorodish FD35-100, WPI, UK) in culture medium. Cells were incubated in OptiMEM containing Fura-2 AM (5 µM) (Molecular Probes, F1201 – 1 mg), the membrane-permeant acetoxymethyl ester form of Fura-2, diluted in DMSO, during 45–60 min at 37 °C. Cells were then washed with OptiMEM and left for 2 additional minutes before recording. For SOCE measurement, free-Ca^2+^ PSS (1 ml) was added and cells were treated by Thapsigargin (5 µM), after a stabilizing time (around 150 s). After total ER-depletion, PSS, with 2 mM CaCl_2_, was added.

Samples were analyzed using either a microscope or a FlexStation. The excitation light source was a 75-W Xenon arc lamp. Excitation light at the two-excitation wavelengths maxima of Fura-2 (340/380 nm) was chopped by a monochromator (Cairn Optoscan, UK). The excitation protocol was a 50 ms excitation at each wavelength every 4 s. Excitation light was directed through a 20× objective with a numerical aperture of 1.4 (Nikon Plan Apo, France). Fluorescence emissions at 510 ± 20 nm were detected by a filter (PMT) placed in the microscope body. Cells were then added in 96-well clear-bottom plates (Corning, USA), at a density of 20,000 cells/well. For the Flexstation, cells were incubated at 37 °C under 5% CO_2_ for 24 h. Ca^2+^ flux was measured with the Fura-2 dye at 5 μM. Cell medium was removed, 200 μl of the dye was added, and then the mixture was incubated for 45 min to 1 h at 37 °C. Prior to the experiment, 100 μl/well of FlexStation buffer for SOCE, free-Ca^2+^ solution) was added. The excitation wavelengths were set at 340 and 380 nm, and the emission was set at 510 nm (auto cutoff: 495 nm). The injection volume was 20 μl/well. Measurements were performed every 4 s. [Ca^2+^]_c_ was calculated as described previously using *in situ* calibration^38^. A Kd of 135 nM was used for these calculations, according to the supplier information on this batch of Fura-2 (Molecular Probes, USA). The physiological saline solution (PSS) or 2Ca solution had the following composition (in mM): NaCl 140, MgCl_2_ 1, KCl 4, CaCl_2_ 2, D-glucose 11.1 and HEPES 10, adjusted to pH 7.4 with NaOH. The Ca^2+^ free solution or 0Ca is a PSS solution without CaCl_2_ and with 1 mM EGTA. The ionomycine was used at 5 µM. High external potassium solutions were prepared by adding 56 mM KCl (K60) or 76 mM KCl (K80) and high external solutions by adding 56 mM NaCl (Na196) or 76 mM NaCl (Na216).

The peak amplitude of the Ca^2+^ TG responses was measured by calculating the difference between the basal and the maximal Ca^2+^ ratio after TG application, in Ca^2+^-free solution. The rising slope of the Ca^2+^ TG responses was determined by linear regression curve fitting 20 sec after TG application and was an estimation of the speed of the Ca^2+^ release by ER. The area of the Ca^2+^ TG responses was determined by integral calculation of the area under the curve, after TG application. The relaxation of the Ca^2+^ TG responses was determined by the half-return time: it is the subtraction of time at the peak minus the time the return at the half of peak value. Peaks and area TG Ca^2+^ responses represented both Ca^2+^ ER release and/or [Ca^2+^]_c_ decrease (through PMCA Ca^2+^ efflux, Na^+^/Ca^2+^ exchanger (NCX) Ca^2+^ efflux or mitochondria Ca^2+^ uptake but not SERCA Ca^2+^ efflux since TG is present).

### Trans-well migration and invasion assays and Wound healing migration assay

Trans-well migration assays were performed as described previously^22^. Trans-well invasion were assessed as trans-well migration assays but membrane was covered with a Matrigel® matrix^39^. Briefly, after 24 h, stationary cells were removed from the top side of the membrane, whereas migrated cells in the bottom side of the inserts were fixed, stained, and counted in five different fields (magnification, ×200). At least three independent experiments were each performed in triplicate. Wound healing migration assays were performed from cell monolayer on 6-well culture plate where wounds were made with a sterile 2mm-wide tip. After wash, cells were treated or not with different drugs. Phase-contrast images of the wound were obtained at the time of scratching and after 12 h. Automatic acquisitions were performed on a Nikon microscope (eclipse ti), coupled to a Nikon camera (DS Qi2). The system includes a cage incubator (Okolab, USA) controlling temperature, levels of CO_2_ and O_2_. Analyses were performed using NIS Element AR software. Brieflly, the analyzes were processed by measuring the area of the injured area to 0 h and 12 h after injury with and without treatment. The injury area before and after the test was measured and compared to the control. The values were plotted as the percentage of the wound closure, with the initial width set to the normalized control.

### Flow cytometry analysis

Cells were incubated at 4 °C with saturating concentrations of CaV1.3 antibody (1:200 Alomone ACC-311) in the dark for 45 min, washed twice with PBS, supplemented with 0.1% Azide-PBS-4% FBS. The same antibody directed against an extracellular epitope of Ca_V_1.3 channel, anti-Ca_V_1.3 (extracellular) antibody (ACC-311 antibody, Alomone) was used for intracellular and extracellular staining. For intracellular staining, cells were washed with cold PBS then incubated for 20 min with 100 μL of Cytofix/Cytoperm™Fixation/Permeabilization Kit (BD Biosciences). After, cells were incubated with a rabbit anti-CaV1.3 antibody for 45 min at 4 °C. Then, cells were washed and incubated with an anti-rabbit coupled to an Alexa Fluor 488 for 45 min at 4 °C. All flow cytometry (FCM) analyses were performed with a minimum of 10000 events using a Gallios flow Cytometer and Kaluza version 1.2 (Beckman Coulter).

### Immunofluorescence microscopy

HCT116 cell line were plated overnight on glasses and subsequently fixed (PBS 1X PFA 4%), permeabilized (Triton × 100 0.1%), blocked (BSA 5%) and incubated with primary rabbit anti-CaV1.**3** (extracellular) antibody (ACC-311, Alomone, 1/200) for 1 h at 4 °C. Glasses are washed (3x) and incubated with a secondary AlexaFluor 488-conjugated anti-rabbit (green color, Invitrogen, Carlsbad, NM, USA, dilution 1/400). Control experiments were performed using only secondary AlexaFluor 488-conjugated anti-rabbit. Acquisitions were performed with a JAI camera (model CV-M4 + CL), with the use of an automated filter wheel coupled to a Leica DMRB fluorescence microscope (Leica Microsystems). Analyses were performed using ImageJ software (NIH, Bethesda, MA, USA).

### Data Availability

The datasets generated during and/or analysed during the current study are available from the corresponding author on reasonable request.

## Electronic supplementary material

Supplemental Method ans Figures
