# Supplementary material for: Ca2+ protein alpha 1D of CaV1.3 regulates intracellular calcium concentration and migration of colon cancer cells through a non-canonical activity
Source: Sci Rep. 2017 Oct 27;7:14199. doi: 10.1038/s41598-017-14230-1 (PMC5660277; doi:10.1038/s41598-017-14230-1)
Supplement: Supplementary file 1 — Supplemental Method ans Figures [file 41598_2017_14230_MOESM1_ESM.doc]

**Ca^2+^ protein alpha 1D**

**of CaV1.3 regulates intracellular calcium concentration and migration of colon cancer cells through a non-canonical activity**

Yann Fourbon^1,8*^, Maxime Guéguinou^1,8*#^, Romain Felix^1,8*^, Bruno Constantin^2,8^, Arnaud Uguen^3,4^, Gaëlle Fromont^1,7,8^, Laurie Lajoie^5^, Christophe Magaud^2^, Thierry Lecomte^5,7,8^, Emmanuel Chamorey^6^, Aurélien Chatelier^2^, Olivier Mignen^3,8^, Marie Potier-Cartereau^1,8^, Aurélie Chantôme^1,8*^, Patrick Bois^2*^ and Christophe Vandier^1,8*^

^1^Inserm, UMR 1069, Université François Rabelais Tours, France.

^2^Equipe ERL 7368 CNRS, Université de Poitiers, France.

^3^Inserm UMR 1078 IFR148, Université de Bretagne Occidentale, France.

^4^CHRU Brest, France

^5^GICC– UMR 7292 Université de Tours, France.

^6^Unité d’Epidémiologie et Biostatistiques (UEB), Centre Antoine Lacassagne, France.

^7^CHRU Tours, France.

^8^From network “Ion channels and cancer-Canceropole Grand Ouest, (IC-CGO) France (www.ic‐cgo.fr).

**Supplemental methods**

**Drugs and Antibodies**

Apamin (SK3 blocker), Forskolin (adenylate cyclase activator), Verapamil (Ca^2+^ inhibitor), Nifedipine (Ca^2+^ antagonist belonging to the dihydropyridine), Bayk 8644 (Ca^2+^ channel activator (L-type)), KB-R7943 (Na^+^/Ca^2+^ exchanger inhibitor (reverse mode), Thapsigargin (a Ca^2+^ ATPase inhibitor), and SEA0400 (Na^+^/Ca^2+^ exchanger inhibitor, A3811, Cliniscience) were added to the PSS or culture media at the concentrations indicated in the figure legends. Besides SEA0400 and all others drugs were purchased from Sigma-Aldrich (St Quentin, France).

**Small interference RNA (SiRNA) transfection assays**

Pre-designed siRNAs (sc-42690, Santa Cruz Biotech) named SiRNAα1D#1 and SiRNA α1D#2 (5’-GAACUCUUCGCUUUCGAAtt-3’) synthetized by Sigma-Aldrich were used to knock-down α1D protein of CaV channels. Pre-designed siRNAs (sc-29234, Santa Cruz Biotech) were used to knock-down calregulin. Sequence of non-targeting siRNA (SiCTL) is described previously ^20^. SiRNAs were delivered to the cells using Lipofectamine^TM^ RNAiMAX transfection reagent (Thermo Fisher Scientific, France) following the manufacturer's protocol. Cells were cultured for 48 hours to obtain optimal silencing of targeted genes.

**Western blot**

To determine α1D protein expression, the cells were washed with PBS and whole-cell lysates were prepared with 5% sodium dodecyl sulphate and protease inhibitor cocktail (Sigma-Aldrich). Ninety micrograms of total cell lysate were resolved by electrophoresis through a SDS-polyacrylamide gel, which was transferred to Hybond™–P, polyvinylidene difluoride (PVDF) membranes (Amersham Pharmacia Biotech, Buckinghamshire, UK). After blocking with 1% BSA in 10 mM Tris (pH 7.4), 150 mM NaCl and 0.1% Tween 20, the filters were probed with specific antibodies. Anti-CaV1.3 (ACC-311, Alomone, 1/200), Anti-Hsp90 (SC-1057, Santa Cruz Biotech, USA, 1/500) or anti-β-actin (A5441, Sigma Aldrich, 1/10000) were used. The proteins were visualized with peroxidase coupled secondary antibodies using the enhanced chemiluminescence detection system, ECL (Pierce® ECL Western Blotting Substrate, Thermo Scientific, France) and a CCD camera (MF ChemiBIS, DNR Bio-imaging Systems, Israël).

To determine NCX1 and NCX3 proteins expression, HTC116 cellular culture were washed with cold phosphate-buffered saline (PBS) and lysed by scraping the cells into a radioimmunoprecipitation assay buffer (in mmol l−1: Tris-HCl 50 pH:8, NaCl 150, EDTA 5, 0.05% Igepal, 1% deoxycholic acid, 1% Triton X-100, 0.1% SDS) containing protease and phosphatase inhibitors (Protease Inhibitor Cocktail, Sigma-Aldrich - PhosSTOP Phosphatase Inhibitor Cocktail, Roche). Soluble cell lysates were denatured 5 min at 37°C in 2× Laemmli sample buffer (in mmol l−1): Tris-HCl 125 pH:6.8, SDS 4%, glycerol 20%, bromophenol blue 0.004%, β-mercaptoethanol 10%). Protein samples (20 and 40 μg), obtained from HTC116 culture, were separated by SDS-PAGE using 8% polyacrylamide gels and transferred to nitrocellulose membranes. Membranes were blocked 1 h 30 in TBS-Tween blocking solution (in mmol l−1: Tris 20 pH : 7.6, NaCl 150 and Tween-20 0.2%) with 5% non-fat milk at room temperature. Blots were then incubated overnight at 4°C with primary antibodies diluted in TBST-5% nonfat dry milk. We used polyclonal anti-rabbit NCX1 or NCX3 (1:200, Alomone Labs, Jerusalem, Israel). GAPDH was probed by mouse monoclonal anti-GAPDH (1:5000, HyTest, Turku, Finlande). Membranes were washed with TBS-Tween three times for 10 min and then incubated for 1 h 30 at room temperature with specific anti-rabbit or anti-mouse horseradish peroxidase-conjugated secondary antibodies (1:5000, Interchim, Montluçon, France). Membranes were revealed with enhanced chemiluminescence (ECL) chemiluminescent substrate (GE Healthcare, Velizy-Villacoublay, France). The results were analyzed by using the GeneGnome Imager (SynGene Ozyme, Montigny - le - Bretonneux, France).

**Cell viability**

Cell viability was determined using the tetrazolium salt reduction method (MTT). Cells were seeded on 24-well plates at a density of 60,000 cells per well and after 4 h drugs were added Measurements were performed in triplicate 24 hours after treatment as described previously ^37^.

**qPCR**

Reverse transcription-qPCR experiments were done according to standard protocols. Briefly, total RNAs were extracted using the Nucleospin RNA II kit (Macherey-Nagel) and used for cDNA synthesis using PrimeScript RT-PCR kit (Takara, Ozyme, France). Quantitative PCR was performed using SYBR® Premix ExTaq™ (Takara) with the LightCycler 480 instrument (Roche Diagnostics, Basel, Switzerland) and with the following parameters: 45 cycles of 10 sec at 95 °C, 30 sec at 60 °C and 20 sec at 72°C. The experiments were performed in triplicate, the amplifications were analyzed with the LC480 software. The analysis of relative mRNA levels was performed using a delta-CT (ΔΔCt) relative quantification model with HPRT as a reference gene. Each value is expressed as fold change relative to the mRNA level of the control value. Amplifications were performed using the sequences described in Table 4.

Table 4 **Primer sequence. F : Forward primer, R: Rreverse primer.**

| Gene | Access no. |  | Primer pairs 5′-3′ | Amplicon size (bp) |
| --- | --- | --- | --- | --- |
| CACNA1D | [NM_000720.3](http://www.ncbi.nlm.nih.gov/entrez/viewer.fcgi?db=nucleotide&id=427197606) | F | AAGCTGAGGACATCGATCCG | 162 |
| (α1D) |  | R | GTCTCCACCAGCACCAGAGA |  |
| CACNB2 | [NM_000723.4](http://www.ncbi.nlm.nih.gov/entrez/viewer.fcgi?db=nucleotide&id=356582343) | F | TATTCCAGCAAACCACCGCT | 181 |
|  | [NM_199247.2](http://www.ncbi.nlm.nih.gov/entrez/viewer.fcgi?db=nucleotide&id=356582350)  [NM_199248.2](http://www.ncbi.nlm.nih.gov/entrez/viewer.fcgi?db=nucleotide&id=356582351) | R | ATCTGTGACCTCGTAGCCCT |  |
| CACNB3 | [NM_000725.3](https://www.ncbi.nlm.nih.gov/entrez/viewer.fcgi?db=nucleotide&id=332634845) | F | ACCAAAGGTACTCCAGCGTC | 101 |
|  | [NM_001206915.1](https://www.ncbi.nlm.nih.gov/entrez/viewer.fcgi?db=nucleotide&id=332634849)  [NM_001206916.1](https://www.ncbi.nlm.nih.gov/entrez/viewer.fcgi?db=nucleotide&id=332634854)  [NM_001206917.1](https://www.ncbi.nlm.nih.gov/entrez/viewer.fcgi?db=nucleotide&id=332634864) | R | GTGGGCACTGAACCAGCTTA |  |
| CACNB4 | [NM_001320722.1](https://www.ncbi.nlm.nih.gov/entrez/viewer.fcgi?db=nucleotide&id=1002819381) | F | GACATTCCGAGCAACTCCCA | 188 |
|  | [NM_001145798.1](https://www.ncbi.nlm.nih.gov/entrez/viewer.fcgi?db=nucleotide&id=224831261)  [NM_001005747.2](https://www.ncbi.nlm.nih.gov/entrez/viewer.fcgi?db=nucleotide&id=224831260)  [NM_001005746.2](https://www.ncbi.nlm.nih.gov/entrez/viewer.fcgi?db=nucleotide&id=224831259)  [NM_000726.3](https://www.ncbi.nlm.nih.gov/entrez/viewer.fcgi?db=nucleotide&id=224831258) | R | TCCCATCAAACCTGTGCTTCA |  |
| HPRT | [NM_000194.2](https://www.ncbi.nlm.nih.gov/entrez/viewer.fcgi?db=nucleotide&id=164518913) | F | TGACCTTGATTTATTTTGCATACC | 102 |
|  |  | R | CGAGCAAGACGTTCAGTCCT |  |

**Statistics**

Statistical analyses have been performed using SigmaStat software (version 3.0.1a, Systat Software, Inc) or StatView software (for Immunohistochemistry analyses, version 5.0, Abacus Concepts, Berkeley, CA). Unless otherwise indicated, data were expressed as mean ± standard error of the mean (n, number of cells from independent experiments). For comparison between more than two means we used Kruskal-Wallis one way analysis of variance followed by Dunn’s or Dunnet’s post hoc tests as appropriate. Comparisons between two means were made using Mann-Whitney tests.

For Immunohistochemistry analyses comparison between groups was performed using the χ2 test and Kruskall wallis ordinal test. Differences were considered significant when p < 0.05.

**Supplemental Figures**


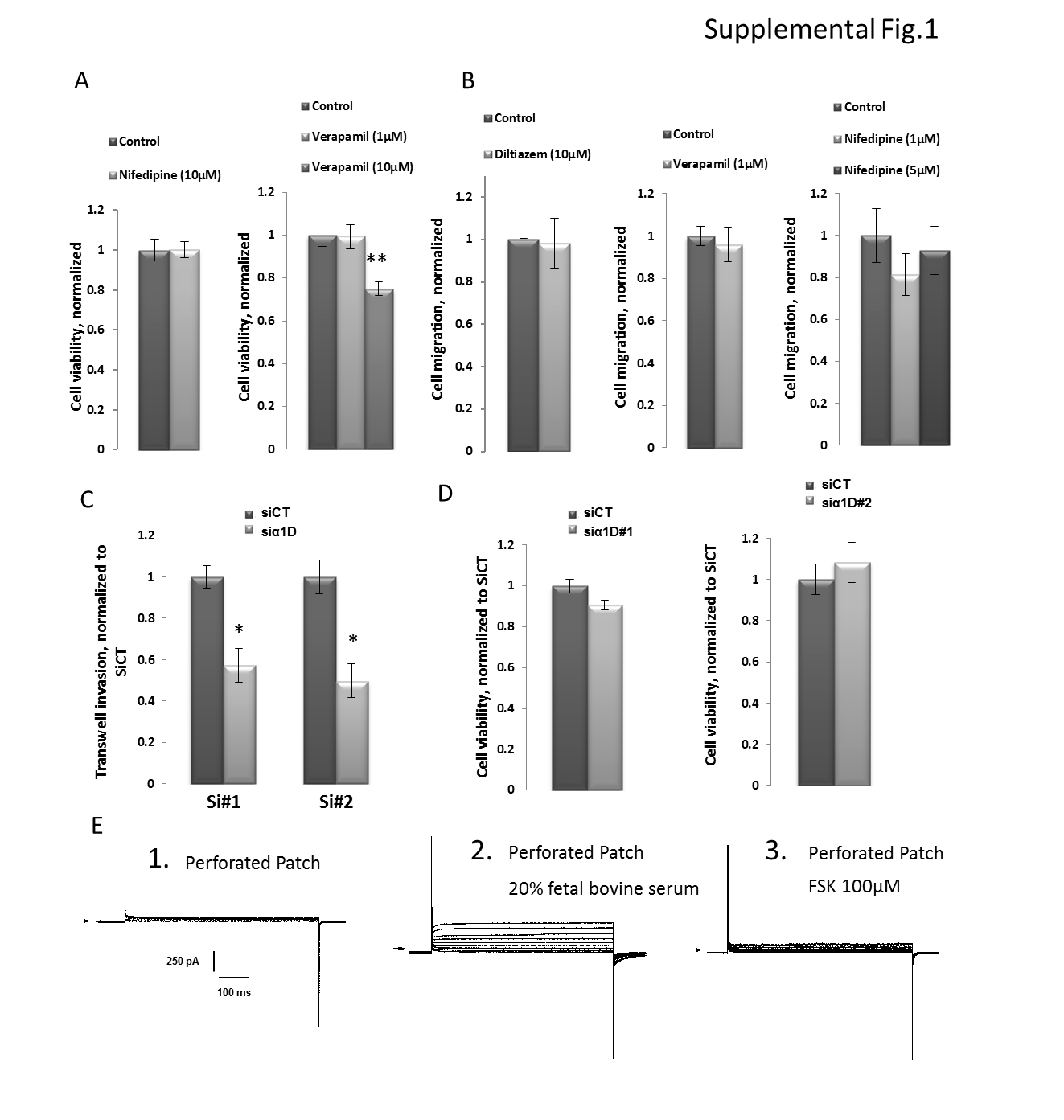


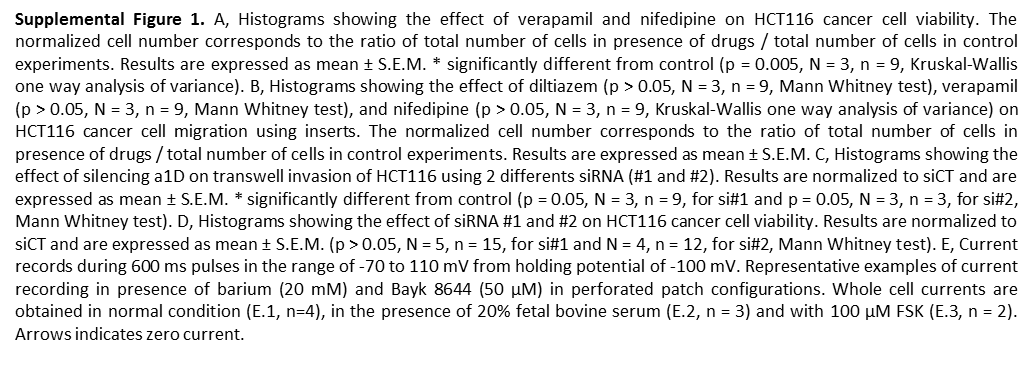


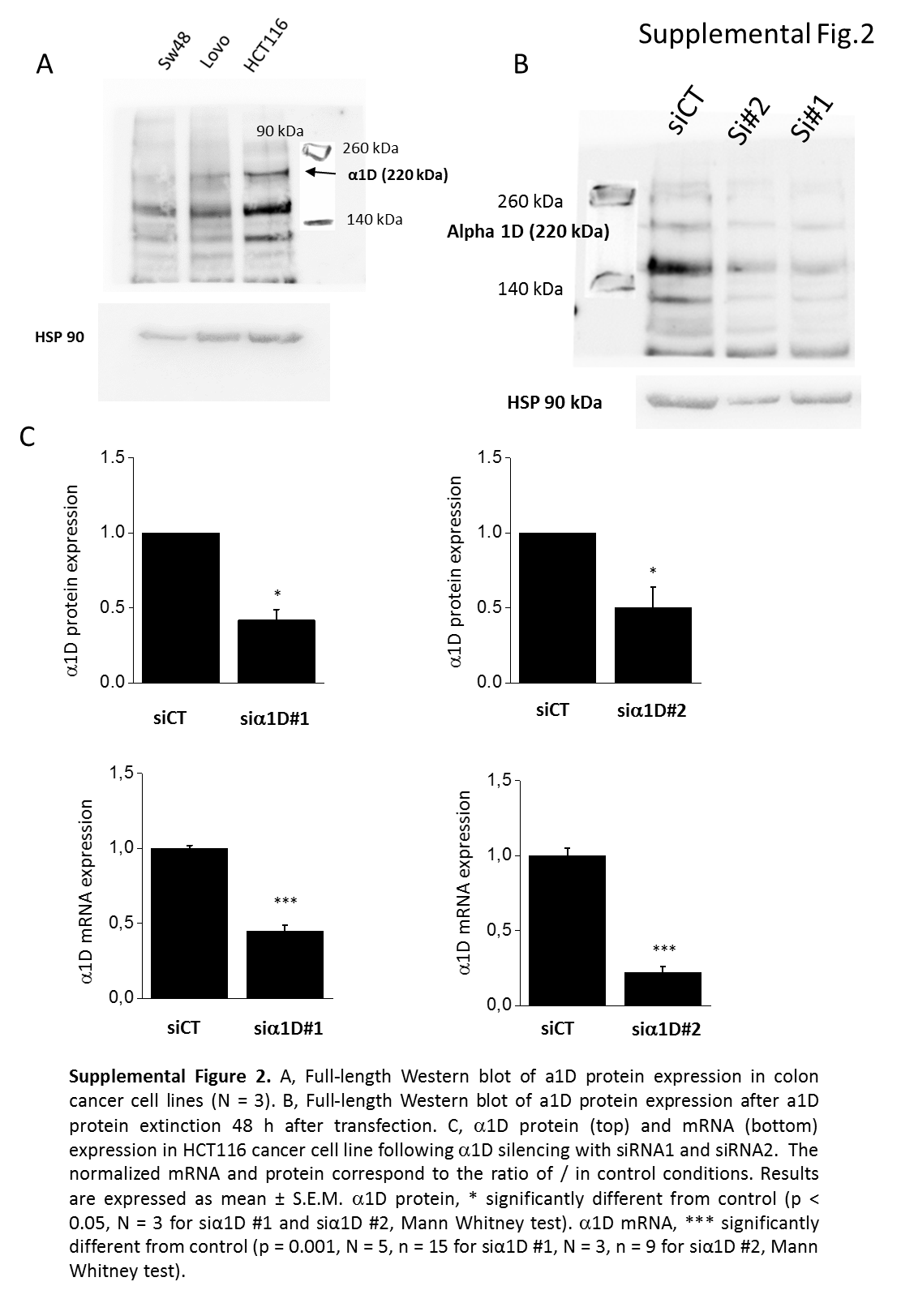


**
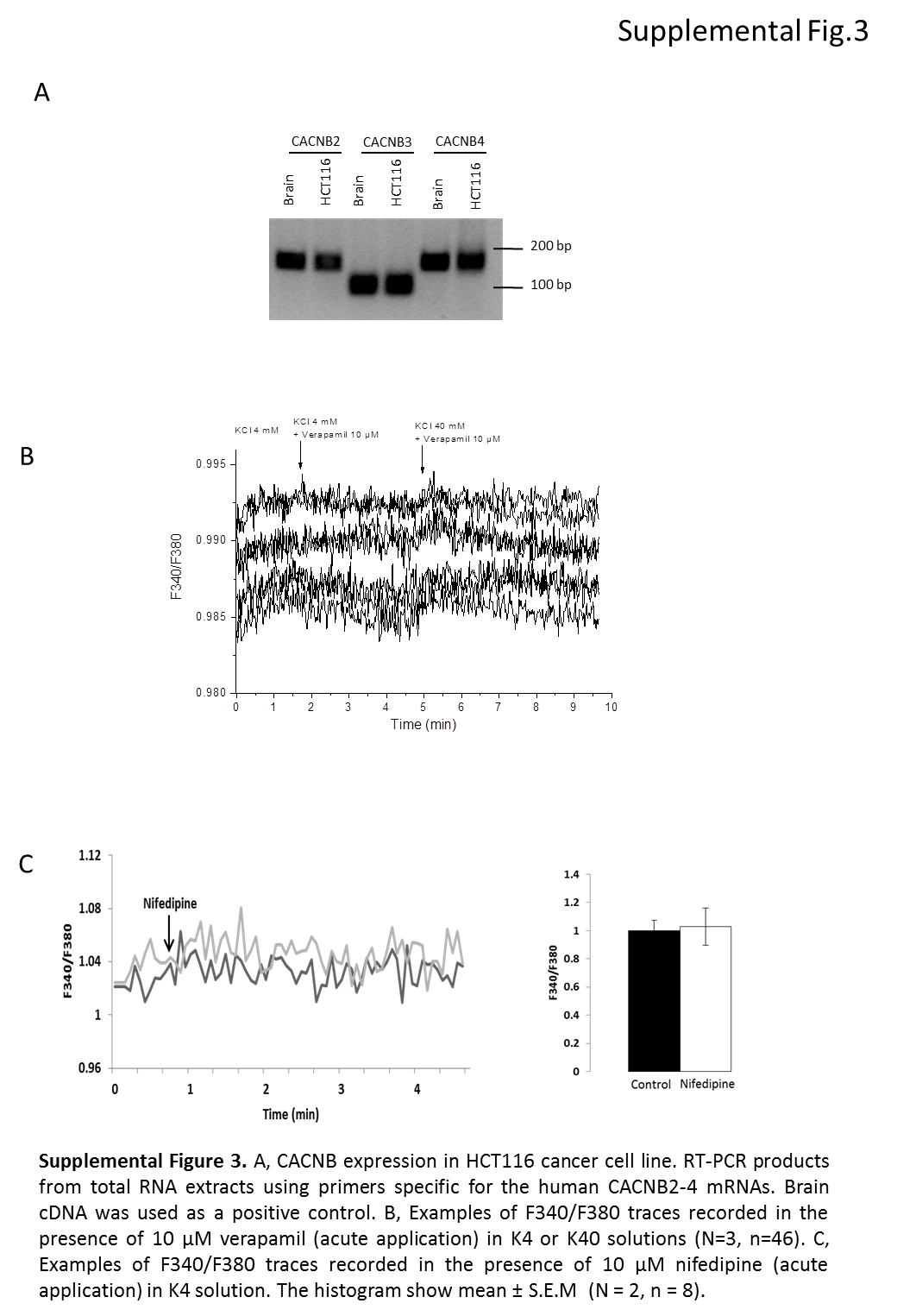
**


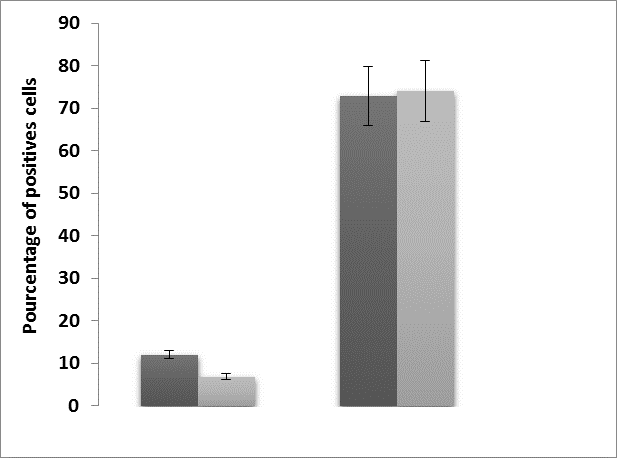


*


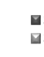


**siCalR**

**siCT**

Supplemental Fig.4

**mCaV1.3**

**tCaV1.3**

**Supplemental Figure 4.** Fluorescence-activated cell sorter (FACS) analysis of α1D proteins in HCT116 cell line with an siRNA directed against calreticulin (siCalR). Permeabilized and not permeabilized cells were stained with an anti-CaV1.3 or an isotype-matched non-binding control mAb. Histograms represent the percentage of cells expressing α1D protein in permeabilized (tCaV1.3) and not permeabilized (mCaV1.3) cells. Results are expressed as mean ± S.E.M. * significantly different from si control condition (p = 0.011, N = 3, Mann Whitney test).

Supplemental Fig.5


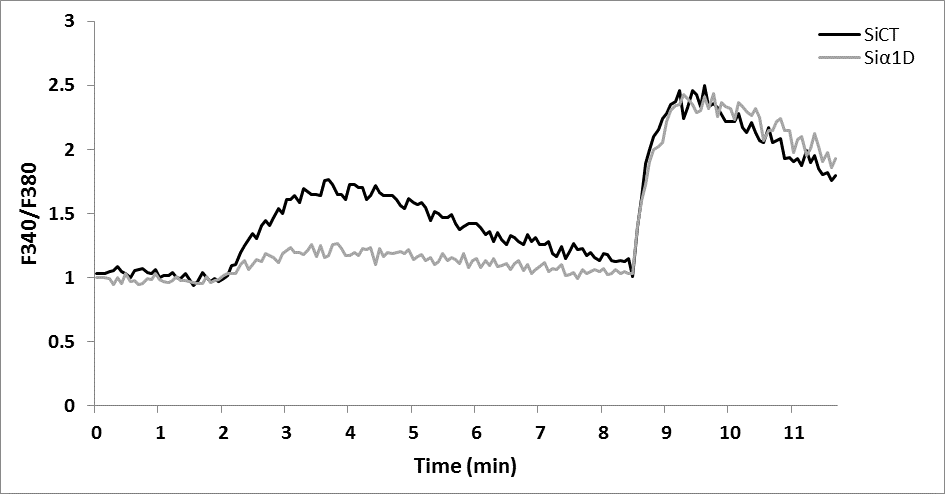

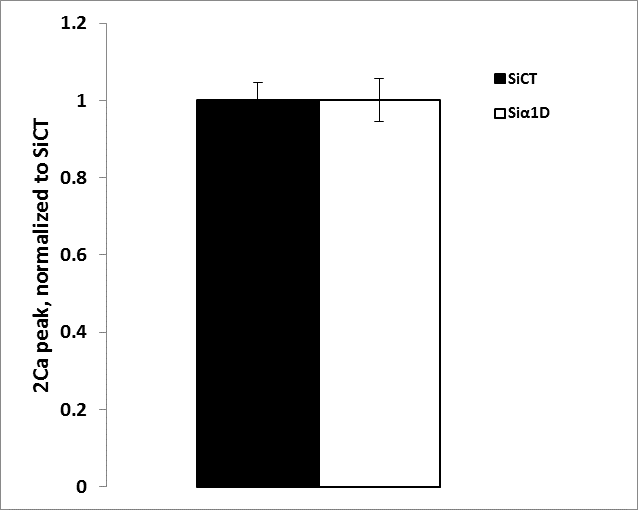


**Supplemental Figure 5.** Silencing α1D with siRNA #1 has no effect on SOCE of HCT-116 cells. Top, typical example of SOCE trace and bottom are means ± SEM. SOCE measurements were performed using the ratiometric fluorescent dye Fura2-AM (5µM). Cells were suspended in PSS Ca^2+^-free solution and treated with thapsigargin (4 µM) to deplete the intracellular store depletion before injection of 2 mM CaCl_2_ solution.

**
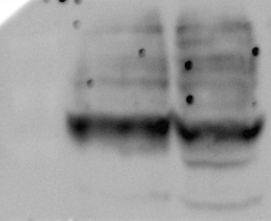

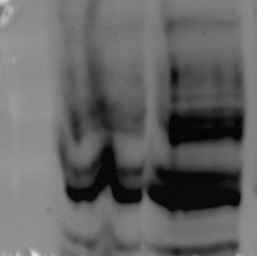

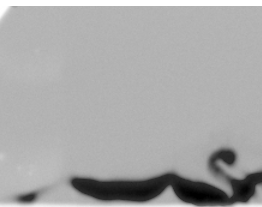

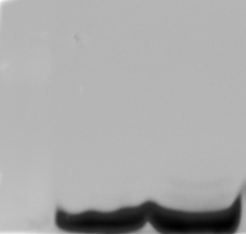
**

Supplemental Fig.6

**20 µg**

**40 µg**

**40 µg**

**20 µg**

**NCX1**

**(SLC8A1)**

**GAPDH**

**GAPDH**

**NCX3**

**(SLC8A3)**

**Supplemental Figure 6.** Full-length Western blot of NCX1 and NCX3 in HCT116 colon cancer cells.

Supplemental Fig.7

**Cell migration, normalized**


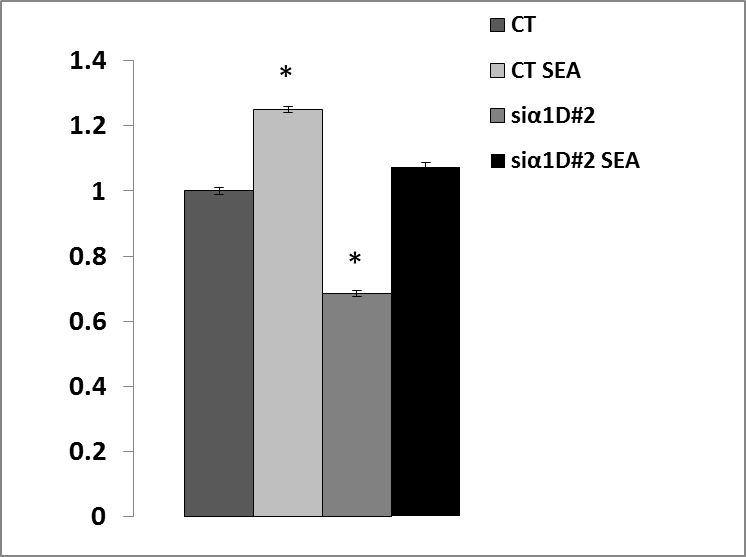


**Supplemental Figure 7.** Effect of silencing α1D in presence or not of 1 µM SEA0400 on migration of LoVo cells (wound-healing cell migration assays). Results are normalized to si control conditions and results are expressed as mean ± S.E.M. * significantly different from control (N=4, n=26, p < 0.001, Kruskal-Wallis one way analysis of variance and post hoc Dunnett's test).
